# Urinary Retention Complications Following Pelvic Floor Reconstructive Surgery: Prevention and Management Strategies Based on Evidence Synthesis

**DOI:** 10.1155/jonm/7634800

**Published:** 2026-05-20

**Authors:** Linlin Zhou, Chunyan She, Shuang Hu, Xue Su, Lubin Liu, Ling Dai

**Affiliations:** ^1^ Department of Obstetrics and Gynecology, Women and Children’s Hospital of Chongqing Medical University (Chongqing Health Center for Women and Children), Chongqing, China

**Keywords:** evidence summary, evidence-based nursing, pelvic floor dysfunction, pelvic floor reconstruction, urinary retention

## Abstract

**Aim:**

To evaluate and summarise the best evidence on the prevention and management of postoperative urinary retention (POUR) following pelvic floor reconstruction and provide an evidence‐based foundation for clinical practice.

**Methods:**

Adhering to the top–down principle of the 6S model of evidence‐based resources, all evidence from domestic and international databases and websites on the prevention and management of POUR after pelvic floor reconstruction, including clinical decisions, evidence‐based guidelines, evidence summaries, systematic reviews, meta‐analyses and expert consensus statements, was systematically searched from the inception of each database to May 9, 2024. Quality assessment, evidence extraction and summarisation were performed by two researchers. This study was registered with the Evidence‐Based Nursing Center of Fudan University (registration number: ES20244725).

**Results:**

Twenty‐one studies were included and used to develop 26 evidence‐based recommendations, which were categorised into five areas: risk assessment and information, preventive measures, early identification, management strategies and follow‐up management. Preoperative evaluation should include the patient’s urination status, postvoid residual (PVR) and risk factors for POUR, without the routine need for urodynamic testing. During surgery, excessive separation of the urethra should be minimised, and the sling or mesh should be kept tension‐free. Catheters should be removed 24–48 h post‐operatively, followed by a timely voiding trial, with retrograde voiding trials recommended. Intermittent catheterisation or indwelling catheterisation is considered the primary treatment option for POUR. If necessary, sling or mesh loosening may be performed, while sling or mesh release should be approached with caution, and urethral dilation is not recommended. A follow‐up assessment of urination status is advised at least once within 6 weeks postsurgery.

**Conclusion:**

These 26 pieces of evidence provide guidance for healthcare professionals in standardising the management of POUR following pelvic floor reconstructive surgery. However, because this evidence comes from different countries, healthcare professionals are encouraged to apply it in specific clinical contexts to optimise patient outcomes. Future research should focus more on risk assessment tools and specific preventive measures for POUR following pelvic floor surgery.

## 1. Introduction

Pelvic floor dysfunction (PFD) is a group of defective and injurious disorders of the pelvic floor supportive tissues caused by various factors, primarily pelvic organ prolapse (POP) and stress urinary incontinence (SUI). These conditions significantly affect the quality of life of women [1, 2], and their burden is likely to become more pronounced as the ageing population increases worldwide. Epidemiological surveys [3, 4] among the Chinese population have reported a 9.6% prevalence of symptomatic POP and an 18.9% prevalence of SUI among adult women. In the United States, the projected number of women undergoing surgery for SUI is expected to increase by 47.2% from 2010 to 2050, and the total number undergoing POP surgery is anticipated to increase from 166,000 to 245,970 [5]. Pelvic floor reconstruction is a primary treatment for severe PFD, typically involving surgical implantation or nonimplantation of mesh materials to repair and reconstruct the normal anatomical structure and function of the pelvic organs, with reported success rates ranging from 78% to 100% [6].

POUR is a common complication following pelvic floor surgery due to the involvement of the bladder and urethra, surgical trauma, and the placement of mesh or graft materials [7, 8]. Yune et al. reported a POUR incidence rate of 26.4% after POP surgery [7], whereas another study, including patients with both POP and SUI, reported rates as high as 48.9% [8]. Severe urinary retention can lead to complications such as urinary tract infections (UTIs), detrusor dysfunction, and even irreversible kidney damage [9]. Patients who develop POUR face prolonged anaesthesia care, longer hospital stays, and a heightened risk of readmission [10]. Recent data [11] have also shown that the average hospitalisation cost for patients with POUR is $100,630, which is significantly higher than for those without POUR, and that post‐discharge expenses can reach $9418. Therefore, early identification and standardised management of POUR after pelvic floor reconstruction are essential.

Currently, evidence regarding the prevention and management of POUR following pelvic floor reconstruction is scattered and lacks specificity. The National Institute for Health and Care Excellence (NICE) guideline on the management of POP and SUI spans 156 pages; however, preoperative assessment of urinary retention following pelvic floor surgery and its brief management are mentioned only in the section on complication management. Several expert consensus statements[13–15] specifically addressing common postoperative complications of pelvic floor surgery focus primarily on treatment strategies for POUR. French clinical practice guideline also includes preventive measures for POUR. Notably, one clinical decision in the diagnostic evaluation of POP is to incorporate preoperative assessment and early detection of urinary retention [17]. Additionally, some studies have described postoperative follow‐up for patients with POUR. Furthermore, Xie et al. [18] summarised evidence on managing voiding dysfunction following anti‐incontinence surgery in women. However, their study population included patients with urinary retention, urgency, and overactive bladder (OAB), whereas our study focuses on patients with POUR. Moreover, some available evidence remains insufficiently comprehensive or specific, including the optimal timing for catheter removal, the choice of voiding trial method, the frequency of intermittent catheterisation, and the criteria for discontinuing intermittent catheterisation and indwelling catheters.

With advances in urogynaecology, evidence on the standardised management of POUR following pelvic floor reconstruction continues to emerge, necessitating supplementation and updates to the relevant evidence. This study is guided by the knowledge‐to‐action (KTA) framework , which comprises two core components: “knowledge creation” and “knowledge application”. Corresponding to the “knowledge creation” phase, this study focuses on POUR as a clinical issue following pelvic floor reconstruction. Through systematic retrieval and rigorous appraisal, it integrates and synthesises the best and most up‐to‐date evidence for the prevention and management of POUR, resulting in this evidence summary. The goal is to facilitate the dissemination and application of relevant evidence among clinical practitioners, provide an evidence‐based foundation for further standardising POUR management, and evaluate the outcomes of practice changes, ultimately achieving a closed‐loop process from knowledge to clinical action. This research followed the evidence summary reporting specifications of the Fudan University Center for Evidence‐based Nursing.

## 2. Methods/Methodology

### 2.1. Problem Establishment

Our research question is as follows: What is the best available evidence for risk assessment, prevention, and intervention measures for urinary retention after pelvic floor reconstruction? To answer this question, we applied the PIPOST model , defining the evidence‐based nursing problem as: “P” (population/evidence application group): female patients diagnosed with POP or SUI requiring pelvic floor reconstruction; “I” (intervention): I1, assessment and identification of POUR; I2, preventive measures for POUR; I3, intervention methods for POUR; “P” (professional implementor): doctors and nurses; “O” (outcome): POUR rate, re‐catheterisation rate, the rate of catheter‐related urinary tract infections (CRUTI), the length of patient’s hospitalisation, and the cost of hospitalisation; “S” (setting for evidence application): the urogynaecology ward or clinics; and “T” (type of evidence): guidelines, clinical decisions, evidence summaries, systematic reviews, meta‐analyses, and expert consensus statements.

### 2.2. Search Methods

Evidence retrieval was conducted according to the pyramid “6S” evidence model, a structured search framework that guides researchers in a top‐down approach, starting from the highest‐quality, pre‐processed evidence sources—such as evidence‐based knowledge bases, evidence summaries, and clinical guidelines—to efficiently retrieve the best currently available evidence [21]. The following databases were searched: BMJ Best Practice, UpToDate, Australian Joanna Briggs Institute (JBI) Healthcare Database, NICE, Scottish Intercollegiate Guidelines Network (SIGN), Agency for Healthcare Research and Quality (AHRQ), Guidelines International Network (GIN), WHO Guidelines Network, Registered Nurses Association of Ontario (RANO), MedPulse, French National Authority for Health (HAS), European Association of Urology (EAU), Society of Obstetricians and Gynaecologists of Canada (SOGC), ACOG, American Urogynecologic Society (AUGS), International Continence Society (ICS), Cochrane Library, PubMed, Embase, Web of Science, Sinomed, Chinese National Knowledge Infrastructure (CNKI), Wanfang, and VIP Database. The search terms used were “pelvic organ prolapse/pelvic reconstructive surgery/pelvic floor dysfunction/colporrhaphy/urogynaecology/urogynecological surgery/vaginal surgery/stress urinary incontinence/sling”; “urinary retention/voiding dysfunction/voiding difficult^∗^/complication^∗^/bladder dysfunction^∗^/urination disorder^∗^”; and “meta‐analysis/systematic review/guideline^∗^/evidence/practical guidance^∗^/consensus/statement^∗^/opinion^∗^” with a search period from the inception of each database up to May 9, 2024.

### 2.3. Literature Inclusion and Exclusion Criteria

The inclusion criteria were as follows: (1) the study subjects were POP or SUI patients who underwent pelvic floor reconstruction; (2) the literature focused on the assessment, prevention, and management of POUR; (3) the types of studies included clinical decisions, guidelines, evidence summaries, systematic reviews, meta‐analyses, and expert consensus statements; and (4) only publicly accessible literature in Chinese or English was considered.

The exclusion criteria were as follows: (1) studies with incomplete information or inaccessible full text; (2) duplicated publications; (3) updated guidelines or articles; and (4) studies that failed the literature quality assessment.

### 2.4. Literature Screening and Quality Evaluation

Literature screening and quality assessment were conducted independently by two authors, Linlin Zhou and Shuang Hu. In cases of disagreement, a third researcher, Chunyan She, was consulted to reach a consensus. Guidelines were evaluated using the “Appraisal of Guidelines for Research and Evaluation II (AGREE II)” updated in 2017 [22]. Systematic reviews, meta‐analyses, and expert consensus statements were assessed based on the criteria established by the JBI Centre for Evidence‐Based Healthcare (2016 version) [23]. There is no internationally recognised tool for evaluating clinical decision‐making and evidence summaries. For such studies, evidence directly sourced from authoritative databases was considered high quality. For evidence obtained from other sources, full‐text information was reviewed, and the original literature was retrospectively assessed using the JBI Centre for Evidence‐Based Healthcare tool.

### 2.5. Evidence Synthesis and Recommendation Level

The synthesis of evidence follows these principles: (1) When the content is consistent, the evidence should be concise, clear, and easily understandable. (2) When the content is complementary, it should be merged into a single piece of evidence based on logical linguistic relationships. (3) When conflicting content arises, evidence should be selected based on the priorities of evidence‐based research, high‐quality evidence, the most recently published authoritative literature, and national guidelines. (4) When the content is independent, the original evidence should be retained.

The included evidence was evaluated and graded using the JBI Evidence‐Based Healthcare Center Evidence Classification and Evidence Recommendation Level System (2014 version) [24]. The system classifies evidence into five levels based on study design. Level 1 represents the highest quality (e.g., systematic reviews of RCTs), followed by quasi‐experimental studies (Level 2), observational studies (Level 3), descriptive studies (Level 4), and expert opinions or basic research (Level 5). For recommendation strength, the JBI Feasibility, Appropriateness, Meaningfulness, and Effectiveness (FAME) framework was used for comprehensive evaluation. This framework assesses not only the evidence quality but also its feasibility in clinical practice, its appropriateness for patients and contexts, its meaningfulness to patient experience and outcomes, and the actual effectiveness of the intervention. Based on this, the evidence are classified as Grade A (Strongly recommended) or Grade B (Weakly recommended) to guide clinical decisions [24].

## 3. Results

### 3.1. Search Results

**FIGURE 1:**
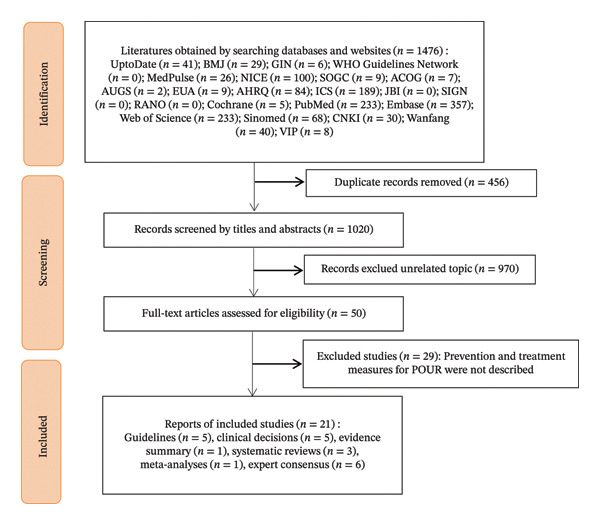
Screening flowchart of included studies.

A total of 1476 articles were retrieved through the search process. Initially, 456 duplicate references were excluded. After reviewing the titles and abstracts, an additional 970 articles unrelated to the topic were removed, leaving 50 articles. Following a full‐text review, 29 articles that did not describe prevention or management measures for POUR were further excluded. Ultimately, 21 articles met the inclusion and exclusion criteria and were included in the analysis. These comprised 5 guidelines [12, 16, 25–27], 5 clinical decisions [17, 28–31], 1 evidence summary [18], 3 systematic reviews [32–34], 1 meta‐analysis [35], and 6 expert consensus articles [13–15, 36–38]. The literature screening process is illustrated in Figure 1, and the basic characteristics of the included studies are detailed in Table 1.

**TABLE 1:** Basic characteristics of the included literature.

Included literature	Year of publication	Country or region	Publishing organisation or author	Database of sources	Target population	Type of evidence
[25]	2017	USA	AUA/SUFU	PubMed	SUI women	Guideline
[26]	2022	Europe	EAU	PubMed	OAB, SUI, MUI women	Guideline
[12]	2019	UK	NICE	NICE	SUI and POP in women ≥ 18 years	Guideline
[16]	2024	France	HAS	PubMed	POP women	Guideline
[27]	2016	Germany, Germany, Switzerland	DGGG/SGGG/OEGGG	PubMed	POP with or without SUI in women ≥ 18 years	Guideline
[28]	2024	/	Nager and Tan‐Kim	UpToDdate	Female SUI undergoing retropubic midurethral slings	Clinical decision
[29]	2024	/	Lippmann and Lukacz	UpToDate	POP and SUI patients undergoing urogynaecologic surgery	Clinical decision
[17]	2023	/	Fashokun and Rogers	UpToDate	POP women	Clinical decision
[30]	2024	/	Mueller	UpToDate	Women with POUR	Clinical decision
[18]	2023	China	Xie et al.	CNKI	Women with SUI undergoing mid‐urethral suspension	Evidence summary
[31]	2023	/	/	BMJ	Women with urinary incontinence	Clinical decision
[32]	2024	USA	Drangsholt et al.	PubMed	SUI patients following midurethral Sling surgery	Systematic review
[33]	2020	UK	Lor et al.	PubMed	Women with urinary incontinence	Systematic review
[34]	2021	China	Xie et al.	PubMed	patients following POP surgery	Systematic review
[35]	2020	China	Li et al.	CNKI	female pelvic floor dysfunction	Meta‐analysis
[13]	2017	USA	ACOG/AUGS	PubMed	POP and SUI patients undergoing mesh surgery	Experts consensus
[36]	2021	China	Chinese Medical Promotion Association Urinary Health Promotion Branch; Chinese Research Hospital Society urological surgery committee	CNKI	POP women	Experts consensus
[14]	2017	USA	IUGA	PubMed	SUI patients undergoing post‐midurethral sling surgery	Experts consensus
[15]	2018	China	Gynecologic Pelvic Floor Group of the Obstetrics and Gynecology Branch of the Chinese Medical Association	Wanfang	POP and SUI patients undergoing pelvic floor reconstraction surgery	Experts consensus
[37]	2019	China	Chinese Medical Promotion Association Urinary Health Promotion Branch; Chinese Research Hospital Society urological surgery committee	CNKI	SUI women	Experts consensus
[38]	2017	China	Gynecologic Pelvic Floor Group of the Obstetrics and Gynecology Branch of the Chinese Medical Association	Wanfang	SUI women	Experts consensus

*Note:* SUFU: Society of Urodynamics, Female Pelvic; NICE: UK National Institute for Health and Clinical Excellence; HAS: French National Authority for Health; DGGG: the German Society of Gynecology and Obstetrics; SGGG: Swiss Society of Gynecology and Obstetrics; OEGGG: the Austrian Society of Gynecology and Obstetrics; AUGS: the American Urogynecologic Society; IUGA: International Urogynecological Association; OAB: overactive bladder; POUR: postoperative urinary retention.

Abbreviations: ACOG, American College of Obstetricians and Gynecologists; AUA, American Urological Association; EAU, European Association of Urology; MUI, Mixed urinary incontinence; POP, pelvic organ prolapse; SUI, stress urinary incontinence.

### 3.2. Results of the Quality Evaluation of the Literature

#### 3.2.1. Quality Evaluation of the Guidelines

Five guidelines [12, 16, 25–27] were assessed, and agreement between the two raters was high. Only four guidelines [16, 25–27] received lower applicability scores, whereas scores in other areas were above 60% for each guideline. Details of the standardised scores for each domain and the overall guideline score are presented in Table 2.

**TABLE 2:** AGREE II scores of the included guidelines.

Included literatures	Standardised score for each field (%)						The overall quality score	Recommend using this guideline
	Scope purpose (%)	Participants (%)	Rigour (%)	Clarity (%)	Applicability (%)	Independence (%)		
[25]	83.33	66.67	73.96	97.22	25	100	5	5
[26]	72.22	75	62.50	94.44	23.83	91.67	5	6
[12]	100	75	82.29	61.11	77.08	100	6	6
[16]	88.89	83.33	61.46	91.67	25	100	6	6
[27]	94.44	66.67	88.54	94.44	33.33	87.5	6	6

*Note:* Domain entries are rated on a 7‐point scale, with 1 being “strongly disagree” and 7 being “strongly agree”. Score rate for each domain = [(actual ratings of the domain by the evaluator – lowest possible rating)/(highest possible rating of the domain − lowest possible rating)] × 100%. Highest possible score = 7 × number of evaluators × number of domain entries, lowest possible score = 1 × number of evaluators × number of domain entries.

#### 3.2.2. Quality Evaluation Results of the Systematic Review and Meta‐Analysis

The study included three systematic reviews [32–34] and one meta‐analysis [35], all of which demonstrated high quality, as detailed in Table 3.

**TABLE 3:** JBI scores of included systematic reviews.

Items	[32]	[33]	[34]	[35]
1. Is the review question clearly and explicitly stated?	Yes	Yes	Yes	Yes
2. Were the inclusion criteria appropriate for the review question?	Yes	Yes	Yes	Yes
3. Was the search strategy appropriate?	Yes	Unclear	Yes	Unclear
4. Were the sources and resources used to search for studies adequate?	Yes	Yes	Yes	Yes
5. Were the criteria for appraising studies appropriate?	Yes	Yes	Yes	Yes
6. Was critical appraisal conducted by two or more reviewers independently?	Yes	Yes	Yes	Yes
7. Were there methods to minimise errors in data extraction?	Yes	Yes	Yes	Yes
8. Were the methods used to combine studies appropriate?	Yes	Yes	Yes	Yes
9. Was there an assessment for possible publication bias?	No	No	No	No
10. Were recommendations for policy and/or practice supported by the reported data?	Yes	Yes	Yes	Yes
11. Were the specific directives for new research appropriate?	Yes	Yes	Yes	Yes

#### 3.2.3. Quality Evaluation Results of Expert Consensus

In this study, six expert consensus studies [13–15, 36–38] were included, and the detailed quality evaluation results are presented in Table​ 4.

**TABLE 4:** JBI scores of included expert consensus.

Items	[13]	[36]	[14]	[15]	[37]	[38]
1. Is the source of the opinion clearly identified?	Yes	Yes	Yes	Yes	Yes	Yes
2. Does the source of opinion have standing in the field of expertise?	Unclear	Yes	Yes	Yes	Yes	Yes
3. Are the interests of the relevant population the central focus of the opinion?	Yes	Yes	Yes	Yes	Yes	Yes
4. Is the stated position the result of an analytical process, and is there logic in the opinion expressed?	Yes	No	Yes	No	Yes	Yes
5. Is there reference to the extant literature?	Yes	Yes	Yes	Yes	Yes	Yes
6. Is there any inconsistence between the ideas presented and the previous literature?	Yes	No	Yes	No	No	No

#### 3.2.4. Quality Evaluation Results of the Evidence Summary

The study included an evidence summary [18] from CNKI, and we tracked the original literature of the reference evidence, of which three guidelines [12, 25, 26] and three expert consensus [14, 37, 38] were included in our study, and one clinical decision was updated; we included the latest version [28] of that clinical decision.

#### 3.2.5. Quality Evaluation Results of Clinical Decisions

Four clinical decisions [17, 28–30] were sourced from UpToDate and one [31] from the BMJ, all of which were included without further scrutiny.

### 3.3. Evidence Synthesis and Generation

A total of 21 studies were included and used to develop 26 evidence‐based recommendations, among which 18 were graded as Level A and 8 as Level B. This evidence was structured into five aspects: risk assessment and information, preventive measures, early identification, treatment strategies, and follow‐up management, as shown in Table 5.

**TABLE 5:** Summary of the best evidence for the prevention and management of urinary retention after pelvic floor reconstruction.

Category of evidence	Content of evidence	Source of evidence	Evidence level	Recommended level
Risk assessment and information	1. Preoperative assessment of patients’ voiding condition and PVR should be conducted to identify potential urinary retention [12, 17, 18, 25, 27, 28, 38].	Multiple expert consensus statements	5b	A
	2. Renal sonography to exclude urinary retention is particularly recommended in patients with high‐grade prolapse [27, 29].	Multiple cohort studies	3b	B
	3. Routine preoperative urodynamic testing is not recommended [12, 14, 17, 25–29, 33].	Multiple systematic reviews of RCTs	1a	A
	4. Urodynamic testing is feasible for further evaluation of bladder function in patient with a history of anti‐incontinence or POP surgery, mismatch of subjective and objective indicators, unclear diagnosis of SUI, mixed incontinence, significant voiding dysfunction, suspected neurogenic lower urinary tract dysfunction, severe POP, and anterior or apical vaginal wall prolapse [12, 16, 17, 25, 26, 28, 29].	Multiple expert consensus statements	5b	B
	5. Assess patients for risk factors for POUR preoperatively, including a history of urinary retention, combined neurological disorders, maximal urinary flow rate < 15 mL/s, preoperative PVR ≥ 150 mL, severe bladder prolapse, comorbid anti‐incontinence surgery, hysterectomy, and rectal prolapse repair, to identify high‐risk individuals for POUR [14–16, 28, 30, 37].	Multiple cohort studies	3b	A
	6. Patients should be routinely informed of the risk of POUR during preoperative communication, especially patients with risk factors [25, 26, 29].	Multiple expert consensus statements	5b	A
	7. Instruct patient with preoperative voiding dysfunction to undergo intermittent catheterisation prior to surgery [30].	Single expert opinion	5c	B

Preventive measures	8. The perioperative use of tamsulosin is recommended to relax the bladder outlet to prevent POUR [16, 29].	Single RCT	1c	B
	9. Surgeon should minimise urethral separation intraoperatively and keep sling or mesh tension‐free to prevent urethral compression at rest [18, 28, 36, 37].	Multiple expert consensus statements	5b	A
	10. It is best to remove the urinary catheter within 24–48 h after the operation [16, 29, 34, 35].	Multiple systematic reviews of RCTs	1a	A

Early identification	11. Early postoperative communication with the patient is recommended to assess whether the patient has significant urinary problems, pain, or other unanticipated events [18, 25, 30].	Multiple expert consensus statements	5b	A
	12. Prompt voiding trail is recommended to identify patients with bladder emptying disorders, and commonly used methods include retrograde voiding trial and spontaneous voiding trial [13, 17, 18, 28, 30].	Multiple expert consensus statements	5b	A
	13. It is recommended to choose retrograde voiding trial, after the patient’s bladder is emptied; 300 mL of sterile saline is instilled retrograde through the catheter or until patients say the bladder has reached its maximum capacity (whichever occurs first); then remove the catheter, and instruct the patient to urinate within 10–15 min. Success is typically defined as a PVR of 100 mL or less or the volume of urine voided is 2/3 or more of the total bladder volume (total bladder capacity = volume of urine voided + PVR), and ultrasound is recommended to determine the PVR [14, 29, 30, 32].	Multiple systematic reviews of RCTs	1a	B

Treatment strategies	14. Patients with postoperative dysuria should undergo relevant examinations to assist in diagnosis and clarify the aetiology, including gynaecological physical examination, ultrasound of residual urine, and urinary ultrasonography, urodynamic testing, and cystoscopy, if necessary [14, 15, 18, 30].	Multiple expert consensus statements	5b	A
	15. Women with persistent voiding dysfunction are recommended to be referred to experienced specialists for diagnosis and treatment [12, 13, 15].	Multiple expert consensus statements	5b	A
	16. For the management of POUR, it is advisable to first consider intermittent catheterisation or indwelling catheterisation [13, 15, 16, 18, 28–31, 36, 37].	Multiple expert consensus statements	5b	A
	17. Patients with adequate cognitive ability, hand coordination, and appropriate body size should be prioritised for intermittent catheterisation, typically performed 4–6 times daily. Catheterisation may be discontinued once the PVR consistently falls below 150 mL or when urine output reaches one‐third of total bladder capacity [13–16, 30].	Case series	4c	A
	18. Patients unwilling to undergo intermittent catheterisation or are unsuitable for it are recommended to have a short‐term indwelling catheter and to undergo a voiding test every 3–4 days until the PVR is less than 150 mL [13, 28, 30].	Multiple expert consensus statements	5b	A
	19. If urinary retention is severe (inability to urinate or only urinate 50–100 mL at a time with a large PVR), and other causes of injury are ruled out, sling or mesh loosening is considered within 2 weeks from the original incision [13, 14, 18, 28, 30].	Multiple cohort studies	3b	A
	20. If sling or mesh loosening still fails to relieve POUR, sling or mesh release may be considered [13, 14, 28, 36, 37].	Multiple cohort studies	3b	B
	21. Women considering sling incision or removal for dysuria should be informed that such procedures may not necessarily resolve voiding problems and carry an increased risk of SUI recurrence or new onset of OAB symptoms [12, 14].	Multiple cohort studies	3b	A
	22. Urethral dilatation is not recommended for the management of postoperative dysuria [14, 18, 28].	Single cohort study	3c	B
	23. Prophylactic antibiotics are not required for either intermittent catheterisation, short‐term or long‐term catheterisation, and antibiotic therapy should be used only in patients with UTI [13, 15, 28, 30].	Single RCT	1c	A

Follow‐up management	24. A telephone follow‐up should be conducted on postoperative Day 2 or 3 to assess the patient’s pain, urination, and bowel movements [29].	Multiple expert consensus statements	5b	A
	25. Patients with POUR can expect to be managed for up to 6 weeks if their residual urine progressively decreases during the follow‐up period [13, 15, 28].	Single RCT	1c	B
	26. Patients should be routinely followed up at least once within 6 weeks after surgery, with long‐term follow‐up advised. The assessment includes routine urinalysis, renal ultrasound, and PVR, as well as the evaluation of symptoms such as urinary difficulties, urinary retention, urgency, etc. [18, 25, 28, 29, 36–38].	Multiple expert consensus statements	5b	A

*Note:* PVR: postvoid residual; POUR: postoperative urinary retention; OAB: overactive bladder.

Abbreviations: POP, pelvic organ prolapse; RCT, randomised controlled trial; SUI, stress urinary incontinence; UTI, urinary tract infection.

In terms of risk assessment and notification of POUR, there are a total of 7 pieces of evidence, of which 1 is grade 1 evidence and 4 is strongly recommended evidence. Multiple expert consensus statements highlight that preoperative assessments should evaluate patients’ voiding conditions and PVR. Several cohort studies have indicated that preoperative screening should include an evaluation of risk factors for POUR. For patients with severe prolapse, renal ultrasound is recommended prior to surgery to rule out urinary retention. Systematic reviews from randomised controlled trials (RCTs) suggest that routine urodynamic testing is not routinely recommended before pelvic floor reconstruction. However, on the basis of multiple expert consensus statements, urodynamic testing can be considered preoperatively for complex cases, such as patients with a history of SUI or POP surgery, SUI with unclear diagnosis, significant voiding dysfunction, or suspected lower urinary tract disorders, to further assess bladder function. Additionally, patients should be routinely informed about the risk of POUR before surgery.

There are three pieces of evidence in the preventive measures of POUR, including two pieces of first‐class evidence and two pieces of strongly recommended evidence. One RCT demonstrated that the prophylactic use of tamsulosin can reduce the risk of POUR. Moreover, a systematic review of RCTs recommended catheter removal 24–48 h post‐operatively. Additionally, several expert consensus statements suggest minimising excessive intraoperative dissection around the urethra, appropriately maintaining tension‐free placement of slings or mesh, and avoiding urethral compression.

In the early identification of POUR, three pieces of evidence are included, with one piece of first‐class evidence and two pieces of strongly recommended evidence. Multiple expert consensus statements emphasise the importance of early postoperative communication with patients to assess for significant voiding difficulties, pain, or other unexpected events. Timely voiding trials should be conducted to identify patients with bladder emptying disorders after catheter removal. On the basis of systematic reviews of RCTs, retrograde voiding trialis recommended as the preferred approach.

There are 10 pieces of evidence for the treatment of POUR, of which 1 is level 1 evidence and 8 are strongly recommended evidence. Multiple expert consensus statements suggest that patients who experience postoperative voiding dysfunction should undergo relevant examinations to aid in the diagnosis and clarify the aetiology. If the issue persists, referral to specialists experienced in managing such conditions is recommended. Intermittent or indwelling catheterisation is considered the initial management approach. Case series studies and expert consensus suggest that for patients with intact cognition, adequate manual dexterity, and suitable body habitus, intermittent catheterisation should be prioritised. For patients who are unwilling or unsuitable for intermittent catheterisation, short‐term indwelling catheterisation may be employed.

Multiple cohort studies indicate that in cases of severe urinary retention—after excluding other causes of injury—sling loosening may be performed, which is typically recommended within 2 weeks via the original incision. If sling or mesh loosening fails to alleviate POUR, sling incision or removal may be considered. Women considering mesh removal to address voiding difficulties should be informed that mesh removal may not necessarily resolve urinary symptoms and may increase the risk of SUI recurrence or the onset of OAB symptoms. A single‐cohort study does not recommend urethral dilation for the management of POUR. Furthermore, on the basis of findings from one RCT, antibiotic therapy should be reserved only for patients diagnosed with UTI.

There is one piece of grade 1 evidence and two strongly recommended pieces of evidence among the three pieces of the follow‐up management of POUR. One RCT indicated that for patients with POUR, if the PVR continues to decline during follow‐up, the management period may be extended to 6 weeks. Multiple expert consensus statements recommend conducting a telephonic follow‐up with patients on the second or third postoperative day to assess pain, voiding, and bowel function. At least one routine follow‐up visit should be scheduled within 6 weeks after surgery, and long‐term follow‐up is also advised.

## 4. Discussion

### 4.1. Screening and Assessment of Risk Factors for POUR

Compared with other surgeries, the unique nature of the surgical site often results in a higher incidence of POUR following pelvic floor reconstruction. Therefore, a thorough preoperative assessment of patients’ urinary function is essential for understanding their voiding status and identifying high‐risk populations for POUR, thereby providing a basis for early intervention. However, this process is frequently overlooked in clinical practice. In accordance with evidence 1–2, a preoperative assessment of the patient’s PVR and renal ultrasound should be performed to confirm the presence of pre‐existing urinary retention. Severe POP may compress the urethra or deform it, leading to abnormal urination and potentially affecting kidney function. Studies [39, 40] have suggested that patients with severe prolapse and a preoperative PVR > 150 mL have an increased risk of developing POUR. Routine preoperative urodynamic testing is not recommended, as it does not correlate with improved patient outcomes [33]. Urodynamic tests are typically conducted by qualified physicians or nurses and involve three components: measurements of the urine flow rate and residual urine, pressure flow studies, and urethral function assessments. The latter two components involve the insertion of a catheter into the urethra and anus, which can cause significant discomfort and carry a risk of UTI. One guideline [12] mentioned that measuring the urine flow rate and residual urine alone is sufficient to identify urinary dysfunction; thus, unnecessary invasive procedures should be minimised as much as possible. However, in specific cases described in evidence 4, experts recommend comprehensive urodynamic testing to further evaluate urethral and detrusor muscle function, thereby guiding surgical decisions.

Evidence 5 indicates that risk factors should be assessed preoperatively. However, there is currently a lack of risk assessment tools for POUR, making stratification of patient risk impossible. Although researchers [41, 42] have explored risk prediction models for POUR following pelvic floor reconstruction, these models have not been recommended in relevant guidelines or clinical decisions, likely because of insufficient validation. With the advent of the age of digital intelligence, we should utilise big data to develop more valuable risk prediction models that are rigorously validated, thereby providing patient‐specific risk estimates for POUR to meet personalised care needs and improve clinical outcomes. Given the increased risk of POUR in patients with pre‐existing urinary retention, evidence 7 mentions that patients should be instructed to perform intermittent catheterisation preoperatively; however, this approach may inadvertently convey negative implications for patients. Hence, further evaluation of this evidence is necessary during evidence implementation.

### 4.2. Implementing Essential Preventive Measures During the Perioperative Period

Preventive strategies are critical for reducing the incidence of POUR following pelvic floor reconstruction. In a study by Chapman et al. [43], patients started tamsulosin 3 days before surgery and continued it for 10 days; the results indicated that the incidence of POUR in the tamsulosin‐treated group was significantly lower than that in the control group. Another meta‐analysis [44] also demonstrated that tamsulosin is more effective than placebo in preventing POUR; however, its effectiveness varies on the basis of surgical site, anaesthesia method, medication management, and catheter use; thus, more RCTs are needed before the drug is widely used in the perioperative period for pelvic floor reconstruction, which is, after all, an off‐label indication for use.

Combined anti‐incontinence surgery or anterior vaginal wall surgery also increases the risk of POUR [45], likely because postoperative oedema around the urethra or tight sling placement alters the angle of the bladder and urethra, leading to bladder outlet obstruction. Consequently, surgeons should minimise urethral separation during surgery and maintain tension‐free slings or meshes to prevent urethral compression at rest. Additionally, the timing of catheter removal postoperatively is associated with the risk of POUR following POP surgery [46]. Although the Infectious Diseases Society of America recommends the early removal of unnecessary catheters to reduce the risk of postoperative UTI [47], two systematic reviews [34, 35] advise against early catheter removal after pelvic floor reconstruction, as it may increase the incidence of urinary retention. To reduce the risk of POUR postpelvic floor reconstruction without increasing the risk of UTI, it is recommended that catheters be removed within 24–48 h post‐operatively, while the optimal time of catheter removal for those patients should be further explored in the future.

### 4.3. Urinary Status Should Be Assessed Promptly Following Catheter Removal and Managed Accordingly

After catheter removal, timely communication with patients is necessary for early identification of urinary problems. Methods for assessing urination include spontaneous voiding trials and retrograde voiding trials. Compared with spontaneous voiding trials, a recent systematic review recommended retrograde voiding trials because of their higher success rates and a lower rate of catheter use upon discharge, while the complication rates for both groups are low and comparable. However, in Chinese clinical practice, spontaneous voiding trials are generally favoured. Pelvic floor reconstructive surgeries are often outpatient procedures abroad, necessitating prompt confirmation of test results; conversely, they are typically inpatient surgeries in China. Retrograde voiding examination may increase patient discomfort and clinical workload, and its feasibility across different regions warrants further investigation. Additionally, recent studies revealed discrepancies in the methods and passing criteria for voiding trials [28, 30]. We recommend considering the patient’s maximum bladder capacity as a standard, as it may be more scientific [48].

Evidence 16–22 outlines specific management evidence for POUR following pelvic floor reconstruction. For early urinary retention, intermittent catheterisation or indwelling catheters are considered. A systematic review revealed that patients with POUR with intermittent catheterisation experienced greater practical benefits [49]. Regardless of the method used, urinary function should be monitored continuously, and catheterisation should be discontinued once appropriate standards are met. It is preferable to perform sling loosening within 2 weeks via the original incision, as the tissue will grow into the sling or mesh over time, making it more difficult for the sling or mesh to be identified and moved [14]. POUR often resolves after sling incision, but this procedure increases the risk of detrusor muscle injury and significantly increases the recurrence rate of SUI, potentially leading to new onset of OAB symptoms [50]. Therefore, thorough preoperative counselling is necessary for patients. Furthermore, sling release or incisions should be carefully selected for patients with preoperative detrusor dysfunction [15]. Currently, the evidence does not support the use of urethral dilation to manage POUR because of the associated risks of UTIs, mesh erosion, and other complications [51]. However, the evidence level of these management strategies requires further improvement.

### 4.4. Standardising the Follow‐Up for POUR After Pelvic Floor Surgery

Transient, delayed or persistent urinary retention may occur in patients after pelvic floor surgery, particularly after anti‐incontinence surgery, where urinary retention is more prevalent [15]. A successful postoperative voiding test does not entirely exclude the risk of urinary retention; thus, short‐term follow‐up for urination is essential. Moreover, the possibility of occult obstruction must be ruled out, as failing to do so may result in missing the optimal window for intervention. In clinical practice, we should establish a long‐term follow‐up mechanism and a follow‐up registration system for patients with mesh to standardise the management of mesh‐related complications. Academic organisations in regions and countries such as the IUGA, Europe, and the United States have successively introduced guidelines or norms for complication diagnosis registration and set up registration platforms [12, 52, 53]. In 2021, a national‐level registration platform for pelvic floor reconstruction was also established in China [54], and an expert consensus on the implant complication registry was subsequently developed [55], laying a strong foundation for the standardisation of the diagnosis and treatment of complications in female pelvic floor surgery in China.

### 4.5. Limitations

Our study has the following limitations: (1) This study included only published Chinese and English literature, possibly biasing the evidence summary results. Hence, broader language inclusion and ongoing evidence updates are advised in future research. (2) There are many surgical procedures and pathways for pelvic floor reconstruction, and the inclusion criteria vary across the original articles, making it challenging to consolidate evidence based on specific surgical methods. Consequently, the suitability of the evidence for each unique situation should be continuously verified in clinical settings. (3) The clinical feasibility of some of the evidence needs to be further investigated, such as the risk assessment of POUR after pelvic floor reconstruction, retrograde voiding test, and prophylactic use of tamsulosin.

## 5. Conclusions

Based on the inclusion of 21 studies, we developed 26 evidence‐based recommendations, which provide guidance for healthcare professionals in preventing and managing POUR after pelvic floor reconstruction, thereby standardising clinical practice. Given the diverse origins of the evidence and the variations in race, concepts, values, health care systems, geography, and culture across different regions, health care professionals are encouraged to apply the evidence based on specific clinical contexts to optimise patient outcomes. Future research should focus more on risk assessment and preventive strategies for POUR following pelvic floor surgery.

## 6. Implications for Nursing Management

The evidence summarised in this study offers an actionable framework for nursing managers seeking to translate evidence‐based recommendations into institutional practice. Based on the 26 synthesised evidence, nurse managers can develop a suite of practical management tools to systematise POUR care. For instance, a POUR risk assessment checklist can be constructed by extracting key risk factors from the evidence (e.g., preoperative PVR, history of urinary retention, concomitant anti‐incontinence surgery). A standardised nursing care pathway can be designed to integrate preoperative risk assessment and risk communication, timing of catheter removal and voiding trials, criteria for intermittent catheterisation and indwelling catheterisation, and follow‐up schedules. Additionally, a set of quality monitoring indicators—such as POUR incidence, indwelling catheter days, and UTI rates—can be embedded into the department’s existing quality assurance framework to enable real‐time tracking and feedback.

Implementing these tools at the unit and organisational level requires careful consideration of contextual factors. Potential barriers extend beyond misalignment between new evidence and existing institutional protocols (e.g., tamsulosin is not routinely used to prevent POUR following pelvic floor reconstruction in clinical practice due to off‐label concerns) and increased clinical workload (e.g., additional preoperative assessments, risk evaluation tasks) to include organisational readiness (e.g., whether the institution has a culture supportive of practice change), infrastructure and training conditions (e.g., availability of portable operating equipment and trained personnel for skills such as retrograde filling testing, PVR measurement, and intermittent catheterisation), and informatics capacity (e.g., whether the electronic health record system can accommodate clinical decision support prompts). Conversely, critical facilitators include visible leadership commitment (e.g., nurse managers actively championing the change); dedicated implementation teams with representation from nursing, medicine, pharmacy, and IT; and alignment with existing regulatory or accreditation requirements (e.g., incorporating POUR metrics into mandatory quality reporting).

To successfully integrate this evidence into routine clinical workflows, a phased implementation approach is recommended. Phase 1 involves translating evidence into user‐friendly tools—for example, converting the risk assessment checklist into a one‐page paper form or an electronic template embedded in admission orders. Phase 2 focuses on workflow integration through informatics: configuring the electronic health record to trigger automatic reminders for risk assessment and risk communication at 24 h preoperatively, and for catheter removal and voiding trials within 24–48 h postoperatively; if a patient meets certain risk criteria or develops POUR, the system prompts nursing staff to implement preventive measures and management strategies. Phase 3 emphasises interprofessional reinforcement: establishing regular multidisciplinary case conferences to review POUR cases, discuss deviations from the pathway, and refine the tools based on real‐world feedback. Phase 4 entails sustainability through measurement: linking adherence to the POUR pathway and associated outcomes to unit‐level performance metrics and incorporating them into nursing competency evaluations or annual goals. By embedding the evidence into structured tools, supported by informatics and reinforced through team‐based learning and accountability systems, nurse managers can transform these recommendations from theoretical guidance into durable components of everyday nursing practice.

## Author Contributions

Linlin Zhou, Ling Dai and Lubin Liu were responsible for the study conception and design. Xue Su and Linlin Zhou performed the literature searching and screening. Linlin Zhou, Chunyan She and Shuang Hu evaluated the quality of literature, conducted evidence analysis, provided summary, and wrote the manuscript. All authors discussed the results and contributed to the final manuscript.

## Funding

This study was funded by Science‐Health Joint Medical Scientific Research Project of Chongqing (grant number: 2023MSXM055) and Chongqing Municipal Health Commission Medical Research Project (grant number: 2025WSJK069). The authors declare that no funds, grants or other support were received during the preparation of this manuscript.

## Conflicts of Interest

The authors declare no conflicts of interest.

## Data Availability

No datasets were generated or analysed during the current study.
